# Effect of oxidative stress and 3‐hydroxytyrosol on DNA methylation levels of miR‐9 promoters

**DOI:** 10.1111/jcmm.14657

**Published:** 2019-09-08

**Authors:** Stefania D’Adamo, Silvia Cetrullo, Rosa Maria Borzì, Flavio Flamigni

**Affiliations:** ^1^ Dipartimento di Scienze Biomediche e Neuromotorie Università di Bologna Bologna Italy; ^2^ Laboratorio di Immunoreumatologia e Rigenerazione Tissutale IRCCS Istituto Ortopedico Rizzoli Bologna Italy

**Keywords:** chondrocytes, epigenetics, hydroxytyrosol, hypomethylation, microRNA, oxidative stress, SIRT1

## 1 INTRODUCTION

Osteoarthritis (OA) is the most common form of arthritis with increasing prevalence. Although it is a multifactorial disease, it is accepted that ageing can induce the onset of OA and has been proposed as the main risk factor of this pathology.^1^ The main reactive oxygen species (ROS) detected in chondrocytes are peroxynitrite (ONOO^−^) and hydrogen peroxide (H_2_O_2_), and when their overproduction is not counter‐balanced by an efficient antioxidant system, the oxidative stress condition occurs that enhances cartilage degeneration and OA.^2^ Furthermore, H_2_O_2_ supplementation has been shown to elicit oxidative stress in chondrocytes.^3^, ^4^ So far, innovative strategies of treatments with no side effects need to be elucidated. For this purpose, diet‐derived natural compounds raised a noteworthy interest due to their preventive and therapeutic action in OA.^5^, ^6^ Hydroxytyrosol (HT), a polyphenol contained in olive oil and derivatives, has been proposed as a fascinating molecule able to reduce oxidative stress‐induced cellular damage and to change epigenetic signature by modulating a microRNA (miR) in chondrocytes.^7^, ^8^ According to our findings, miR‐9 results to be overexpressed under chondrocyte exposure to H_2_O_2_ and miR‐9 dysregulation under TGF‐β1‐dependent ROS increase has been reported in other cell models,^9^, ^10^ thus confirming its susceptibility to redox state and oxidative stress. However, the priming mechanism by which oxidative stress and HT could trigger these modulations is still lacking. Indeed, the molecular key underlying regulation of miR expression in OA is not completely clear and needs further investigation. In humans, miR‐9 is transcribed from three independent genomic loci mapping to chromosomes 1q22 (MIR9‐1), 5q14.3 (MIR9‐2) and 15q26.1 (MIR9‐3). Our present work sought to clarify this aspect by studying DNA methylation of the three miR‐9 promoters in response to H_2_O_2_ and HT treatments in C‐28/I2 chondrocytes.

## 2 MATERIALS AND METHODS

### 2.1 Cell cultures and treatments

C‐28/I2 is a human cell line representative of primary chondrocytes^11^ that has been used for deeper molecular studies to provide mechanistic explanations to the findings of previous work carried out on human primary chondrocytes.^7^ Cells, grown in DMEM medium supplemented with 10% foetal bovine serum, were incubated in the absence or presence of 100 µmol/L H_2_O_2_ for 2 hours; 100 µmol/L HT (Cayman Chemical) was added 30 minutes before H_2_O_2_. The concentration of HT was chosen on the basis of a published study,^12^ and previous experiments reported in our published manuscripts^3^, ^7^, ^13^ have confirmed the efficacy of this concentration in protecting chondrocytes from cell death with lack of toxicity. To assess the effects of modulation of methylase activity on miR‐9 transcription, in a separate series of experiments increasing doses of 5′‐azacytidine (5′Aza; 1‐50 mmol/L) (Sigma‐Aldrich) were added to cells 24 hours before collection.

### 2.2 Cell transfection

C‐28/I2 cells were seeded in 6‐well plates at a density of 2.5 × 10^5^ cells/well in medium without antibiotics. The next day cells were transfected with ON‐TARGETplus Human Sirt1 siRNA (25 nmol/L) or ON‐TARGETplus non‐targeting pool (25 nmol/L) (Dharmacon) by Lipofectamine® RNAiMax Reagent in Opti‐MEM® Medium (Life Technologies) according to manufacturer's instructions and incubated for 48 hours before collection.

### 2.3 Nucleic acid isolation, bisulfite conversion and methylation‐specific PCR

Total cellular RNA and genomic DNA were extracted with 700 µL TRIZOL (Invitrogen), according to manufacturer's instructions. Human Methylated & Non‐methylated DNA Set (Zymo Research, Irvine, CA, USA) was used to provide negative and positive controls. 500 ng of sample and control DNA was treated with sodium bisulfite using the EZ DNA Methylation Kit (Zymo Research, Irvine, CA, USA) according to the manufacturer's protocol. Six pairs of methylation‐specific primers were designed by the online MethPrimer software^14^ and purchased by Invitrogen (miR‐9‐1 meth forward AGGTAGAGGTTTTTTTAGTTTCGTC and reverse AACCTTTCCTCTCTCTTTAAATCG; miR‐9‐1 unmeth forward GGTAGAGGTTTTTTTAGTTTTGTTG and reverse AACCTTTCCTCTCTCTTTAAATCAC; miR‐9‐2 meth forward TTGTTAGAAGAAAAATGTAGGTAAAGAC and reverse CCTACTACCCGAACAACGAC; miR‐9‐2 unmeth forward TTAGAAGAAAAATGTAGGTAAAGATGT and reverse CCTACTACCCAAACAACAAC; miR‐9‐3 meth forward TTTGTTTATTTTTTTTGGTTTTTCG and reverse CTCTCGACTCCTCTAACTCTTACGA; miR‐9‐3 unmeth forward GTGTTTGTTTATTTTTTTTGGTTTTTT and reverse TCCTCTCAACTCCTCTAACTCTTACA). Primers were annealed at 53°C. Platinum™ Taq DNA Polymerase (Thermofisher) was used according to the manufacturer's protocol.

### 2.4 cDNA synthesis and Real‐Time PCR

RNA pellets were treated with DNAse (DNA‐free, Ambion) and quantified by using RiboGreen RNA quantitation reagent (Molecular Probes). MicroRNA reverse transcription was conducted with TaqMan MicroRNA RT kit (Life Technologies), and qPCR was performed with TaqMan Universal Mastermix (Life Technologies) following kit instructions. Mature miR quantification was performed by using TaqMan MicroRNA Assays for miR‐9 and U6 snRNA (internal control), according to manufacturer's recommended protocols.

### 2.5 Western blotting assay

Proteins were separated on 10% SDS polyacrylamide gels, transferred to nitrocellulose membranes (Amersham), and probed with anti‐β‐ACTIN (Sigma‐Aldrich) and anti‐SIRT1 (Santa Cruz Biotechnology) primary antibodies at 4°C overnight.

After washes, membranes were incubated with horseradish peroxidase‐conjugated anti‐mouse (Santa Cruz Biotechnology) IgG for 1 hour. The chemiluminescent signals were detected using an ECL system (Luminata^™^ Crescendo, Millipore).

### 2.6 Statistical analysis

Data are reported as mean ± standard deviation (SD). Means were compared with GraphPad Prism5 statistical software (GraphPad Software, Inc). Differences were considered statistically significant at *P* < .05.

## 3 RESULTS

### 3.1 MiR‐9 expression is increased by H_2_O_2_‐induced promoter demethylation

**Figure 1:**
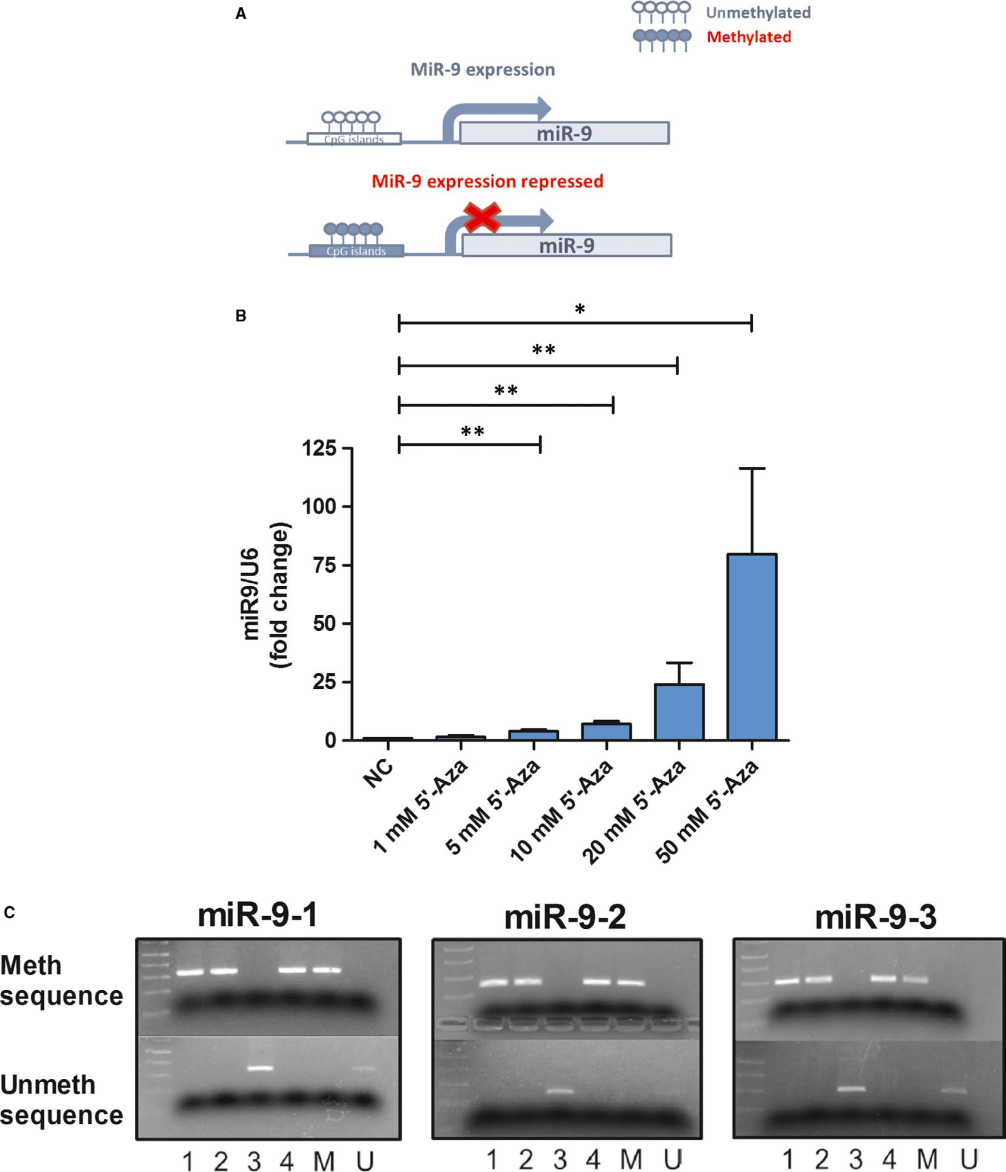
MiR‐9 promoters are influenced by 5′‐Azacytidine, Hydroxytyrosol and Hydrogen peroxide treatments. A, Schematic drawing of the hypothesis that miR‐9 expression is dependent on methylation status of its promoters. B, qRT‐PCR analysis of miR‐9 levels in 5′‐Aza‐treated cells (n = 4 independent experiments). C, MSP analysis for methylated and unmethylated sequences of miR9‐1, miR9‐2 and miR9‐3. 1 (non‐treated cells), 2 (HT‐treated cells), 3 (H_2_O_2_‐treated cells), 4 (HT + H_2_O_2_‐treated cells), M (universal methylated DNA), U (universal unmethylated DNA). Values are expressed as mean ± SD, *P < .05, **P < .01

Our previous study^7^ showed that miR‐9 levels increase after treatment with H_2_O_2_ and decrease with HT. In order to evaluate if miR‐9 expression could be influenced by methylation status of its promoters in our cellular model (as drawn in Figure 1A), 5′‐Aza, a DNA methyltransferase (DNMT) inhibitor, was used. The levels of miR‐9 increased after 5′‐Aza treatment in a dose‐dependent manner (Figure 1B). Therefore, the status of CpG islands surrounding promoters of miR‐9 genes is important for the regulation of gene expression.

Promoter methylation levels of miR‐9‐1, miR‐9‐2 and miR‐9‐3 were assessed in response to HT and/or H_2_O_2_ by using methylation‐specific PCR (MSP). As shown in Figure 1C, levels of miR‐9 methylation were decreased in all three promoters of cells treated with H_2_O_2_ and, on the contrary, reestablished after pretreatment with HT. From a qualitative point of view, no difference in the methylation status among the three different promoters has been observed.

### 3.2 *SIRT1* silencing determines demethylation of miR‐9 promoters

**Figure 2:**
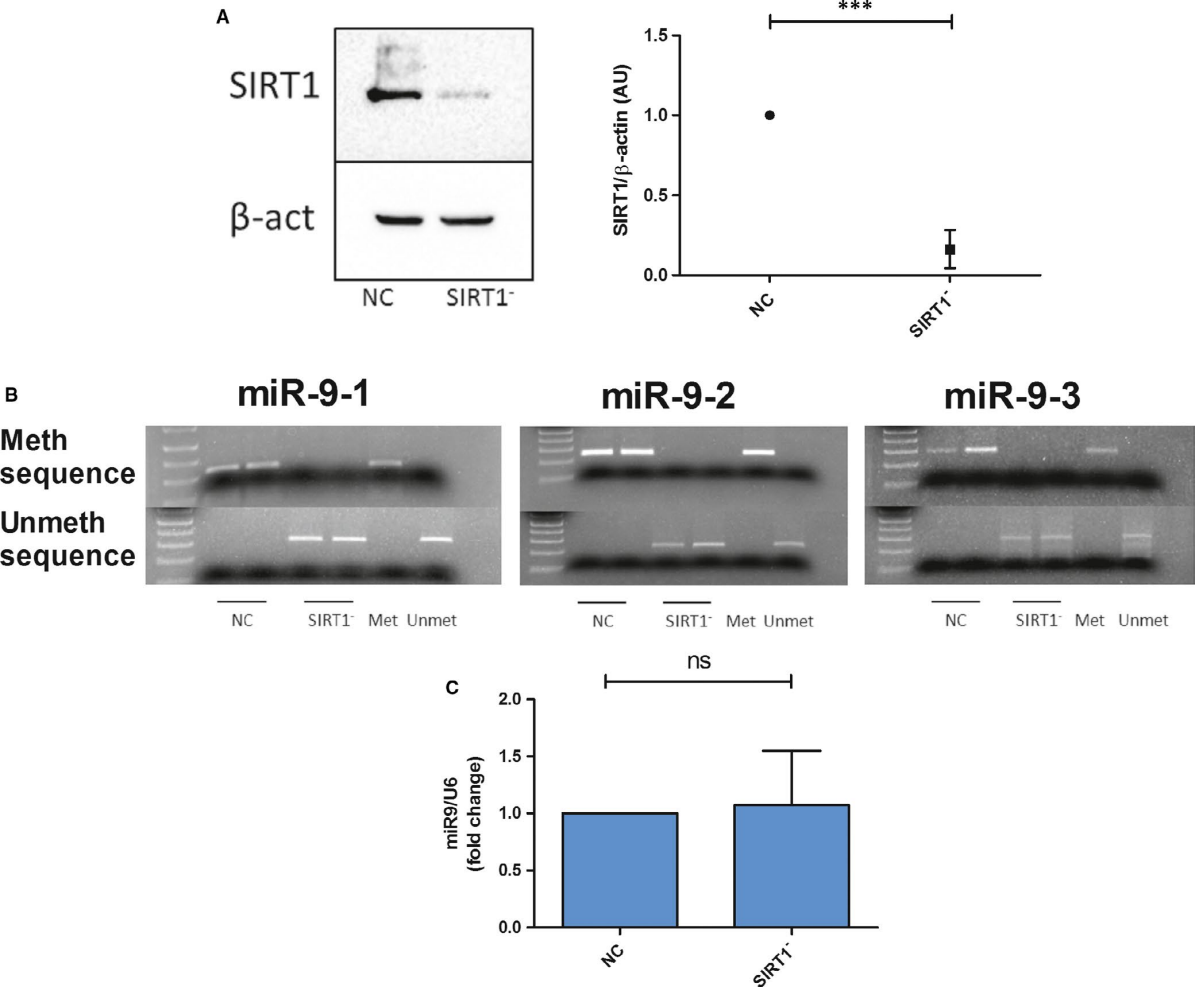
MiR‐9 promoters are demethylated by *SIRT1* silencing without influencing gene expression. A, Western blotting analysis of SIRT1 and β‐ACTIN. Representative images and relative quantifications are shown (n = 4 independent experiments). B, MSP analysis for methylated and unmethylated sequences of miR9‐1, miR9‐2 and miR9‐3. C, qRT‐PCR analysis of miR‐9 levels in *SIRT1*‐silenced cells (n = 4 independent experiments). Values are expressed as mean ± SD, ***P < .001

*SIRT1* has been reported as a genuine target of miR‐9 and SIRT1 levels decreased in response to H_2_O_2_‐induced oxidative stress.^7^ To determine whether SIRT1 could modulate methylation of miR‐9 promoters in a negative feedback loop, C‐28/I2 cells were depleted of *SIRT1* by RNA interference. Protein samples were immunoblotted with SIRT1 antibody to test the transfection outcome (Figure 2A). Then, sample DNA was extracted and analysed by MSP. As shown in Figure 2B, *SIRT1* knockdown changes methylation status of promoters by hypomethylating all three of them. However, we did not observe a corresponding increase in miR‐9 expression in *SIRT1*‐silenced cells (Figure 2C). Thus, *SIRT*1 knockdown by siRNA transfection or H_2_O_2_ treatment can demethylate the promoters, though only H_2_O_2_ treatment is able to modulate miR‐9 expression in response to methylation status of CpG islands.

## 4 DISCUSSION

In our previous work, we demonstrated that HT, a polyphenol found in olives and derivatives, can prevent oxidative stress‐induced cell death and autophagy dysfunction by modulating miR‐9 availability and its cognate target *SIRT1*. Thus, miR‐9 has been identified as a crucial factor orchestrating the molecular response to H_2_O_2_ and HT in chondrocytes.^7^, ^13^ Dysregulated levels of miR‐9 in OA patients have been published,^15^ and besides SIRT‐1, other targets associated with OA pathogenesis have been reported, including MMP‐13^16^ and monocyte chemo‐attractant protein 1‐induced protein 1 (MCPIP‐1).^17^

Nevertheless, the fuse triggering the variations of miR expression was unknown. A genome‐wide DNA methylation study performed in OA cartilage identified miR‐9 as an OA susceptibility gene among other factors.^18^ To explore whether our treatments could influence miR‐9 expression by modifying methylation status of CpG islands surrounding the three promoters of miR‐9 genes, we treated the cells with the DNMT inhibitor 5′‐Aza and detected a dose‐dependent increase in miR‐9 levels. Furthermore, all three miR‐9 promoters were shown to be hypomethylated in cells treated with H_2_O_2_ and hypermethylated in cells treated with HT alone or both. Taken together, these results suggest that these treatments modulate miR‐9 expression by exerting opposite effects on the promoter methylation status, with oxidative stress reducing and HT rescuing and sustaining the hypermethylation of CpG islands. Since no methylation differences among the three promoters have been highlighted, we could speculate that all the three genes contribute to the expression levels of miR‐9.

Since miR‐9 reduces its direct target *SIRT1*, as demonstrated by luciferase assay,^7^ we investigated whether, in turn, SIRT1 could be implicated in the modulation of miR‐9 levels in a negative feedback loop. However, miR‐9 promoter hypomethylation induced by *SIRT1* silencing through RNA interference did not correspond to an increase in miR‐9 expression. Thus, demethylation of miR‐9 promoters can favour but per se may not be sufficient to promote miR‐9 expression. It may be hypothesized that miR‐9 expression requires the involvement of some transcription factors, triggered upon oxidative stress or 5’‐aza‐induced general hypomethylation, but not following just *SIRT1* silencing that may elicit hypomethylation restricted to miR‐9 promoters. If previous work^7^ elucidated the role of this miR in the H_2_O_2_‐promoted cell death and in the protective effect of HT in chondrocytes, these new findings provide the upstream mechanism influencing the variations of miR‐9 expression. The identification of a miR able to address the cell fate in response to a protective and/or stress agent opens novel perspectives in the field of molecular therapy for degenerative diseases, such as OA. Indeed, a better understanding of the interaction of different epigenetic levels in OA pathogenesis, including promoter methylation status, miR expression and transcriptome changes, could be useful to prime further investigations for a miR‐based strategy with nutraceutical support in the treatment of this disease.

## CONFLICT OF INTEREST

The authors have declared that there is no conflict of interest.

## AUTHOR CONTRIBUTION

SD designed the experiments. SD and SC performed the experiments. SD, SC, RMB and FF analysed and interpreted the data. SD, SC, RMB and FF contributed in writing and approving the manuscript.

## Data Availability

The datasets used and/or analysed during the current study are available from the corresponding author on reasonable request.
